# Mind the cap: Detecting degradation impurities in synthetic mRNAs

**DOI:** 10.1016/j.omtn.2025.102701

**Published:** 2025-09-17

**Authors:** Daniel Nielsen, Maria Victorova, Timothy R. Mercer, Seth W. Cheetham

**Affiliations:** 1 Australian Institute for Bioengineering and Nanotechnology, University of Queensland, Brisbane, QLD, Australia; 2 BASE Facility, University of Queensland, Brisbane, QLD, Australia

## Main text

The 5′-cap on messenger RNA (mRNA) is essential for translation, stability, and innate immune avoidance. Methods to accurately quantify 5′-cap integrity are essential to ensuring the quality of mRNA medicines.

A recent *Molecular Therapy Nucleic Acids* article comprehensively characterized mRNA 5′-cap integrity and its degraded products. The impact of 5′-cap degradation products on mRNA translation and the cellular immune response was also evaluated, with implications for the manufacture and quality control of mRNA medicines.^1^

Hutchinson et al. present an extensive analysis of 5′-cap degradation impurities arising during *in vitro* transcription (IVT) and downstream processing of mRNA therapeutics. Using orthogonal ion-pair reverse phase ultra-performance liquid chromatography-mass spectrometry (LC-MS) with electrospray ionization workflows, the authors show that Cap-1 structures, commonly used in mRNA medicines, are chemically vulnerable to degradation under physiological and manufacturing conditions. The degraded products reduce protein expression levels but do not trigger innate immune activation.^1^ These findings underscore the importance of considering 5′-cap degradation as a critical quality attribute (CQA) during the manufacture of mRNA medicines.

As the mRNA field expands beyond vaccines into new therapies, cancer vaccines, immunotherapies, and gene editing tools, the quality control of mRNA molecules is becoming increasingly important to ensure potency, safety, and ultimately, commercial success. The 5′-cap structure found in higher eukaryotes (Cap-1, N7-methylguanosine linked to a 2′-O-methylated nucleotide^2^^,^^3^) is essential for efficient ribosomal recruitment, host RNA distinction against pathogen RNA genome, and prolonged intracellular stability. Accordingly, emphasis has been placed on minimizing uncapped species during manufacturing due to their immunostimulatory potential.^4^^,^^5^ However, less attention has been paid to subtle chemical degradation of the cap structure itself, which may erode therapeutic efficacy even in fully capped preparations.

This study focuses on overlooked impurities by characterizing degradation products resulting from stress conditions (pH, temperature, and metal ions), standard experimental and even unstressed conditions, revealing their impact on expression without immune activation. The findings suggest a more nuanced view of mRNA stability, where cap integrity is due to both synthesis conditions and analytical artifacts related to RNase digestion and LC-MS analysis.

**Figure 1:**
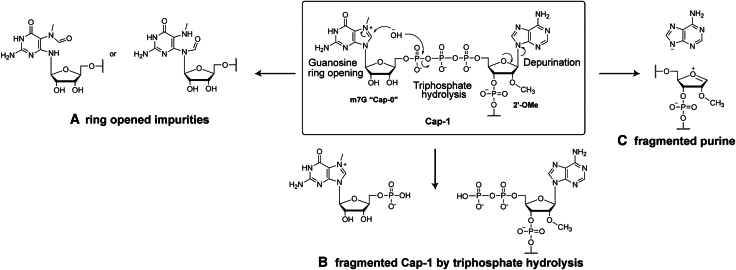
Cap-1 degradation (A) ring opening hydrolysis of imidazole ring in m7G, (B) fragmented Cap-1 following triphosphate hydrolysis, and (C) purine fragmentation.

Hutchinson et al. employed two orthogonal LC-MS approaches, RNase H and RNase T1 digestion workflows, to analyze Cap-1-modified firefly luciferase (FLuc) mRNA. Both methods enabled high-resolution profiling of capped, uncapped, and chemically modified 5′ mRNA ends. Notably, a +18 Da mass shift corresponding to imidazole ring opening of the m7G cap by hydrolysis was identified as a major impurity (Figure 1A). This hydrolytic degradation has previously been described but not in the context of mRNA translation.^6^ Other degradation pathways, including triphosphate bridge hydrolysis (Figure 1B) and depurination (Figure 1C), were also detected. The degradation was also observed for both synthetic cap analogs and capped full-length IVT mRNAs, indicating that degradation can impact both Cap-1 monomers and larger mRNA constructs with cap structures incorporated. Studies revealed that degradation was minimal in water but significantly accelerated in the presence of MgCl_2_ and spermidine, which are both common components of IVT reactions, together with elevated pH and temperature. All factors, combined and individually, were shown to accelerate Cap-1 degradation even under unstressed conditions. This is concerning, given that IVT reactions often operate at pH 7.9–8.5 and 37°C–40°C,^7^ and suggests that standard manufacturing practices can cause cap degradation.

To assess the impact of degradation on mRNA translation, the team engineered a panel of model FLuc mRNAs with varying levels of cap degradation and uncapped impurities. In A549-Dual cell assays, uncapped impurities were shown to reduce expression and increase activation of innate immune sensors. In contrast, Cap degradation products caused a dose-dependent decline in protein expression, without detectable activation of immune-responsive interferon regulatory factor or nuclear factor κB pathways. This difference likely results from the lack of immunostimulatory triphosphate ends on degraded caps and potential processing by the Dcp2 decapping enzyme cleaving m7-GpppN cap structure to release m7-GDP leaving a non-immunogenic 5′-monophosphate mRNA,^8^ leading to its further degradation by conserved 5′→3′ exoribonucleases.^9^ From a therapeutic perspective, this distinction is crucial. While uncapped RNAs represent a dual threat, both inefficacious and immunogenic, they are easily detected in standard capping assays. Cap-degraded species, however, potentially fly under the radar of routine immune assays yet compromise potency. This work therefore expands the definition of what constitutes a CQA in mRNA therapeutics as cap-degraded molecules may not give rise to immediate safety concerns but rather pose a challenge to efficacy and dose consistency.

The study also found that the detection of some degradation can be artifactual, arising during sample handling or LC-MS analysis itself. The authors show that high pH in analytical buffers and elevated liquid chromatography column temperature can inadvertently induce cap degradation, resulting in overestimating actual impurity levels. They propose the use of reference standards and minimized digestion times to correct for these effects, adding an important methodological note to the field’s growing reliance on LC-MS-guided quality control.

Together, the implications of the presented study for mRNA therapeutic manufacturing are significant. Current IVT protocols may try to minimize exposure to conditions that promote degradation. Formulations and storage buffers should be evaluated not only for RNA stability but also for the long-term integrity of the cap. Cap analog and reagent suppliers may also need to certify degradation profiles during quality control analysis. Since even capped mRNAs can degrade over time into non-functional forms, routine stability testing should also include cap integrity assays and not focus solely on full-length RNA assessments.

Looking ahead, this study opens multiple avenues for future exploration. First, it will be important to determine whether cap degradation impacts intracellular pharmacokinetics beyond initial translation, for example, by influencing RNA decay rates or subcellular localization. Second, since current cap analogs are vulnerable to hydrolytic attack, chemical innovation may yield more stable analogs resistant to ring opening and triphosphate cleavage. For example, cap analogs with phosphodiester backbone modifications limit decapping enzyme activity and self-cleavage of the phosphodiester backbone at physiological pH.^10^ Third, *in vivo* studies will be needed to validate whether the observed expression losses translate into reduced therapeutic effect or altered biodistribution.

More broadly, this study underlines the need for comprehensive strategies in all stages of nucleic acid therapeutics development. The success of mRNA medicines will rely on a thorough understanding of the molecular identity of mRNA therapeutics as well as degradation products. As analytical capabilities evolve, so too must our quality specifications of molecular identity and functional integrity.

In summary, Hutchinson et al. offer an important contribution to the molecular pharmacopeia of mRNA therapeutics. Their study reveals that the 5′-cap, long regarded as a binary feature (present or absent), exists on a chemical spectrum that shapes the performance of mRNA drugs. As the field moves into more demanding therapeutic applications, this detailed understanding of cap stability and degradation will be essential. Future advances on cap chemistry, IVT process design, and analytical method development will all benefit from the foundational insights provided here.

## Acknowledgments

We acknowledge the following sources of funding and support: the Australian Government Research Training Program (RTP) Scholarship to M.V., 10.13039/501100000925National Health and Medical Research Council (GNT1161832 to T.R.M.), the 10.13039/501100000923Australian Research Council (DE230100036 to S.W.C.), the Medical Research Future Fund (MRFCRI000063 and MRFNCRI000089 to S.W.C. and T.R.M.), the National Collaborative Research Infrastructure Strategy (NCRIS) to T.R.M. and S.W.C., and 10.13039/501100020111Therapeutic Innovation Australia (TIA) to T.R.M. and S.W.C. BASE is supported by TIA. TIA is supported by the Australian government through the National Collaborative Research Infrastructure Strategy (NCRIS) program.

## Declaration of interests

The authors declare no competing interests.
